# Changes in chromosome territory position within the nucleus reflect alternations in gene expression related to embryonic lineage specification

**DOI:** 10.1371/journal.pone.0182398

**Published:** 2017-08-02

**Authors:** Maciej Orsztynowicz, Dorota Lechniak, Piotr Pawlak, Beata Kociucka, Svatava Kubickova, Halina Cernohorska, Zofia Eliza Madeja

**Affiliations:** 1 Department of Genetics and Animal Breeding, Poznan University of Life Sciences, Poznan, Poland; 2 Veterinary Research Institute, Brno, Czech Republic; Michigan State University, UNITED STATES

## Abstract

Loss of totipotentcy in an early embryo is directed by molecular processes responsible for cell fate decisions. Three dimensional genome organisation is an important factor linking chromatin architecture with stage specific gene expression patterns. Little is known about the role of chromosome organisation in gene expression regulation of lineage specific factors in mammalian embryos. Using bovine embryos as a model we have described these interactions at key developmental stages. Three bovine chromosomes (BTA) that differ in size, number of carried genes, and contain loci for key lineage regulators OCT4, NANOG and CDX2, were investigated. The results suggest that large chromosomes regardless of their gene density (BTA12 gene-poor, BTA5 gene-rich) do not significantly change their radial position within the nucleus. Gene loci however, may change its position within the chromosome territory (CT) and relocate its periphery, when stage specific process of gene activation is required. Trophectoderm specific CDX2 and epiblast precursor NANOG loci tend to locate on the surface or outside of the CTs, at stages related with their high expression. We postulate that the observed changes in CT shape reflect global alternations in gene expression related to differentiation.

## Introduction

Gamete fusion at fertilisation marks the beginning of preimplantation development. The newly formed, totipotent zygote retains capacity to form all cell types (both embryonic and extraembryonic). The dynamic interplay of nuclear architecture, genome organisation and epigenetic reprogramming provides the basis for directing embryonic cell fate. As a result, at the blastocyst stage, two independent cellular lineages emerge: pluripotent inner cell mass (ICM) and differentiated trophpectoderm (TE).

Another unique feature of preimplantation development is a drastic genome wide rearrangement of the nuclear architecture. Recent studies suggest that the three-dimensional (3D) spatial position of genomic regions during interphase may be an important factor linking chromatin architecture with gene expression regulation. Such changes have been documented during development, reprogramming and disease progression [1–4]. Limited data exist linking chromosome organisation with lineage commitment of embryonic cells. Being interested in mechanisms regulating embryonic cell fate and pluripotency maintenance in non-rodent mammals (with bovine embryos serving as a model), we have followed the 3D changes in chromosome organisation through the distinctive stages of preimplantation development.

Our understanding of early development mostly relies on data obtained from extensive studies in mice. It was shown that the key regulatory factors are universal, yet their timing of activation and/or mutual regulation may vary depending on the species. The first differentiation events result from cellular exposure to different microenvironment (the inside/outside model) when upon compaction, blastomeres acquire polarised apical surface [5–7]. The development of the apical domain at the morula stage precedes the action of TE specific transcription factors: CDX2, EOMES, ITGA7, CDH3 and ELF5 [8–11]. In mouse, CDX2 is present in heterogenous levels in all blastomeres at the morula stage, where it overlaps with the ICM and the epiblast (EPI) specific markers such as OCT4 and NANOG [rev by [12]]. OCT4 is placed in the centre of the gene network that preserves pluripotency and is essential to maintain both the ICM and the embryonic stem cells (ESCs) [13]. It is initially expressed in all blastomeres of the mouse embryo and becomes restricted to the ICM 3.5 days post insemination (dpi). At the molecular level the interactions between TEAD4, YAP protein and mutually repressive signalling of CDX2 and OCT4 result in the establishment of the TE/ICM lineages. Prior to the transition from the ICM to the epiblast further regulatory transcription factors are activated such as NANOG and SOX2 [14, 15]. In mouse epiblast, NANOG proves to be essential for transition from a totipotent (unstable) to the pluripotent (stable) state. During this transition NANOG also becomes crucial for the reactivation of the paternal X chromosome [16–18].

Recent studies by our group indicate that the process of lineage segregation in bovine embryos resemble the mouse model [19]. At the blastocyst stage (9dpi) OCT4 and NANOG signals separate to different cell populations within the ICM and CDX2 becomes TE specific [19–21]. The level of molecular interactions might be different in non-rodent mammals, as Berg et al. (2011) indicated that CDX2 does not directly repress OCT4 expression, but it is required for TE maintenance at the later stages. Such observations proved to be true also for humans, primates, pigs, horses and rabbits [20, 22–25].

Assuming the important role of nuclear architecture in the process of gene expression regulation, we have chosen to follow the changes in 3D distribution bovine chromosomes (*bos taurus* chromosomes, BTA) 5, 12 and 23, which make bovine embryos an ideal model for such studies. They differ in size, gene content and contain loci for *NANOG*, *CDX2* and *OCT4* genes respectively. Interphase chromosomes are arranged in chromosome territories (CTs) which occupy specific regions of a nucleus [26]. Depending on the cell type, CTs are arranged according to their gene density or DNA content [27]. Studies indicate that within the nucleus gene-dense CTs are mostly found internally and gene-poor CTs peripherally [28]. The specific position of gene’s locus within the CT, may be an additional factor regulating its expression. Loci may localise at the surface, the interior or may loop out of CTs, promoting an environment that is permissive or repressive for local gene expression. It still remains unclear whether the elevated levels of transcription cause an increased frequency of chromatin looping, or whether high levels of gen activity promote a certain chromosome region to be extruded from its CT. Studies suggest that gene rich domains, with ubiquitous gene expression patterns have tendency to locate outside their CT more often than gene poor regions. However, it was observed that the β-globin locus looped out from its CT despite the lack of robust transcriptional activity. The observed looping was correlated with relocation to a transcriptionally repressive compartment [29, 30].

Studies of mouse embryos show that the timing of nuclear architecture reconstruction coincides with the period of epigenetic reprogramming, loss of totipotency and initiation of the first cell fate decisions [31]. The detailed investigation of higher order chromatin arrangements during major bovine embryonic genome activation (EGA) revealed its close correlation with the establishment of non-radial CT arrangement [2]. Prior to the EGA, gene density of a chromosome does not correlate with its radial distribution (distance to the nuclear centre). After the EGA gene poor chromosomes occupy more peripheral regions of the nucleus than gene rich chromosomes. Thus, it may be concluded that in order for EGA to take place, the early embryo must remodel its initially transcriptionally oppressive chromatin status into permissive totipotent configuration. At the 8-cell stage of mouse development, Ahmed at al. (2010) detected dispersed and uniform chromatin architecture, suggesting that extensive epigenetic remodelling occurs after the EGA. This open chromatin structure was largely devoid of blocks of compact chromatin, being similar to the pluripotent ICM/EPI cells. Relatively dispersed chromatin was also noted for undifferentiated ESCs, suggesting that the distinct nuclear architecture may be inherited from the embryo, rather than acquired during stem cell derivation. Global chromatin organisation may provide the structural basis in the nucleus that distinguishes pluripotent cells from tissue-restricted progenitors. Study of chromatin structure, dynamics and organization emerge to be crucial to the understanding of the regulatory processes of self-renewal and pluripotency maintenance within the embryo. Microscopic visualization of DNA in ESC nuclei show diffuse staining indicative of a generally open chromatin state [31, 32]. Consistent with a decondensed and permissive chromatin state, pluripotent and totipotent cells exhibit higher chromatin mobility [4, 33].

Relying on the existing data we have hypothesised that lineage commitment should be marked by global changes in CT radial positioning and its 3D structure, that allows for transcriptional activity of regions containing the key regulatory genes. The overall conclusion from our work specifically points to the important role of mutual interactions between chromosome size, CT shape and its location within the nucleus. Large CTs such as 5 and 12 locate at the periphery. However, this unfavourable positioning which may restrain the access of transcription machinery to certain gene regions, may be overridden by the specific behaviour of gene locus. Similarly to the observations made in the somatic cells specific gene regions may temporarily relocate to the surface or outside of its CTs allowing for transcription to commence

## Materials and methods

### Ethics approval

All procedures were performed in accordance with the “Act on the protection of animals used for scientific purpose” of the Republic of Poland, which complies with the European Union Legislation for the protection of animals used for scientific purposes. According to these regulations ethics approval was not required, as the biological material (ovaries) was collected upon animal slaughter in abattoir (Meat Plant Biernacki Ltd., Poland) or bought from commercial Artificial Insemination and Animal Reproduction Station (bull semen). *In vitro* obtained bovine embryos were of preimplantation stages (up to day 9 of development) which are not subjects of the animal protection act, as according to Art 2.1 of the “Act on the protection of animals used for scientific purpose” the embryonic form of animals that are under 1/3 of development do not require ethics approval.

The total number of embryos used for each experiment is presented in Table 1.

**Table 1:** Number of embryos used for each experimental setting.

DEVELOP. STAGE/ANALYSES	3D RECONSTRUCTION EMBRYOS/NUCLEUS			IMMUNOSTAINING			GENE EXPRESSION		
	CDX2	OCT4	NANOG	CDX2	OCT4	NANOG	CDX2	OCT4	NANOG
ZYGOTE	23/46	25/49	-	3	4	-	80	80	80
2–4	18/29	19/30	-	22	20	-	80	80	80
8–16	10/36	10/34	9/50	10	9	8	80	80	80
MORULA	10/71	10/60	5/102	6	5	9	32	32	32
BLASTOCYST	10/60	10/60	2/20	15	18	16	16	16	16

Unless stated otherwise all reagents were supplied by Sigma Aldrich. Fluorescent signals were visualized using Zeiss Axiovert 200M laser scanning confocal microscope.

### In vitro production of bovine embryos (IVP)

IVP was done as previously described by Madeja et al. [19]. For the purpose of IVP bovine cumulus oocyte complexes (COC) were collected from abattoir ovaries. COCs were aspirated from 2–6 mm ovarian follicles, selected for *in vitro* maturation (IVM) according to their morphology and subjected to 24h incubation in TCM 199 medium supplemented with 1mg/ml fatty acid free bovine serum albumin (fafBSA), 0.05 mg/ml gentamicin, 0.022 mg/ml Na-pyruvate, 2.2 mg/ml NaHCO_3_ and hormones (5 UI/ml hCG, 10 UI/ml PMSG, Intervet) at 39°C in humidified atmosphere with 5% CO_2_. Insemination was done with certified artificial insemination bull semen at a concentration of 1x10^6^/ml. Crioprotectants were removed by centrifugation (2x) in standard IVF-Sperm-Talp medium supplemented with 4 mg/ml fafBSA. Sperm capacitation was induced by PHE (penicillin, hypotaurine and epinephrine). After 20h of gamete coincubation (39°C, humidified atmosphere, 5% CO_2_) cumulus cells were mechanically removed and the presumptive zygotes were transferred in groups of 25 to 35μl to *in vitro* embryo culture (IVC) drops covered with mineral oil and cultured at 39°C in humidified atmosphere with 5% CO_2_, 5% O_2_, 90% N_2_. The basic IVC medium was based on synthetic oviduct fluid (SOF) supplemented with essential (BME) and nonessential (MEM) amino acids and 4mg/ml fafBSA. At 3 dpi only cleaved embryos of no less than 3–4 cells were placed in fresh IVC culture drop and left in culture until collection. Zygotes were collected 20hpi, 2–4 cell embryos 32-48hpi, 8–16 cell stage embryos 56-72hpi, morulae at 116-128hpi (5dpi), blastocysts at 8dpi and hatched blastocysts (HBl) at 9dpi. Timing was chosen after [34].

### Fluorescent in situ hybridisation, 3D-FISH

After collection the embryos were briefly washed in PBS at 37°C and fixed at room temperature for 10 min in 4% paraformaldehyde (PFA) as described by Koechler et al. [2]. *Zona pellucida* was removed by incubation in 0.1N HCl (up to 1 min), followed by double incubation in 0.05% Triton X-100/0.1% BSA/PBS (10 min each) and permeabilisation (1h in 0.5% Triton X-100/0.1% BSA/PBS). Embryos were again incubated in 0.1N HCl (up to 1 min), subjected to series of 10 min washes (2x in 0.05% Triton X-100/0.1% BSA/PBS, 2x in 0.1% Triton X-100/0.1% BSA/2xSSC) and incubated at least for 48h in 50% formamide/2xSSC. After the over-night (o/n) incubation embryos were washed twice for 10 min in 0.1% Triton X-100/0.1% BSA/2xSSC, equilibrated for 10 min in 0.05% Triton X-100/0.1% BSA/PBS and permeabilised for 1h in 0.5% Triton X-100/0.1% BSA/PBS/0.02% RNaze A. After short wash in 0.05% Triton X-100/0.1%BSA/PBS embryos were incubated for 2 min in 0.1N HCl. Finally, to remove the 0.1N HCl solution, the embryos were washed once in 0.05% Triton X-100/0.1% BSA/PBS and twice 0.1% Triton X-100/0.1% BSA/2xSSC for 10 min. Before hybridization the embryos were incubated for at least 48h in 50% formamide/2xSSC.

Probes pairs consisting of CT + locus specific probes were analysed in separate hybridization procedures as described by Koehler et al. [2] with modifications. *In situ* hybridization was performed in 1.5 ml tubes (Eppendorf). The total volume of 4.5 μl painting probe (about 0.5 μg) was added to the tube containing 4 μl of previously precipitated locus specific probe (1 μg of locus specific probe in 4 μl 50% formamide, 10% dextran sulphate, 2xSSC, 1% Tween 20) and briefly mixed. For each experiment a total number of 15 embryos was transferred to hybridization mixture consisting of painting and locus specific probes. To prevent evaporation, the drop containing molecular probes and embryos was covered with mineral oil. After 2-3h equilibration at 37°C the embryos and the molecular probes were denatured for 8 min at 76°C. Hybridization was carried out for 72 h at 37°C in a humidified atmosphere. To remove the hybridization mixture the embryos were washed twice for 10 min in 0.1% Triton X-100/0.1% BSA/2xSSC. Finally the embryos were incubated twice for 10 min at 0.1× SSC/0.1% BSA. Single embryos were transferred to poly-L-lysinated (1mg/ml) 18-well “μ-slides” (Ibidi GmbH) containing 8 μl of Vectashield Mounting Medium-DAPI (1.5 μg/ml; Vector Laboratories) and stored at + 4°C.

#### Chromosome painting and locus specific probes

Whole chromosome painting probes for BTA5, BTA12 and BTA23 were obtained by laser microdissection and labelled with Spectrum Orange-dUTP (Abbott Laboratories) by DOP–PCR. Locus specific probes (*NANOG*—CH240-164H1, *CDX2*—CH240-301P20, *OCT4*—CH240-470G19) were obtained from BAC clones (CHORI-240) and labelled with Spectrum Green-dUTP by nick translation (Abbott Laboratories).

#### Confocal microscopy and imaging

Confocal analyses were performed on Zeiss Axiovert 200M laser scanning confocal microscope. FISH images (Z-stack) were collected using 100x/1.4 oil immersion objective lens for high resolution images. The size-step of Z-stacks (pixel size of 0.09 μm to obtain stacks of 8-bit grey scale image of 1.024 x 1.024 pixels) were 0.5 μm for zygotes, 2–4 cell stage, 8–16 cell stage and 0.4 μm for embryos at the morula and the blastocyst stage. Series of images were processed by AxioVision Rel. 4.8 programme (Zeiss, Germany) and analysed and reconstructed using NEMO software, a tool designated for gene and chromosome territory distribution analysis from 3D-FISH experiments [35]. 3D reconstructions were created using ImageJ (https://imagej.nih.gov/ij/) or ICY 1.7.3.0 (http://www.bioimageanalysis.org/) software. Confocal images/sections of immunolabelled embryos were taken every 4.0 μm and the 3D reconstructions were done with Zeiss LSM software.

#### 3D reconstruction and image analysis

**Fig 1:**
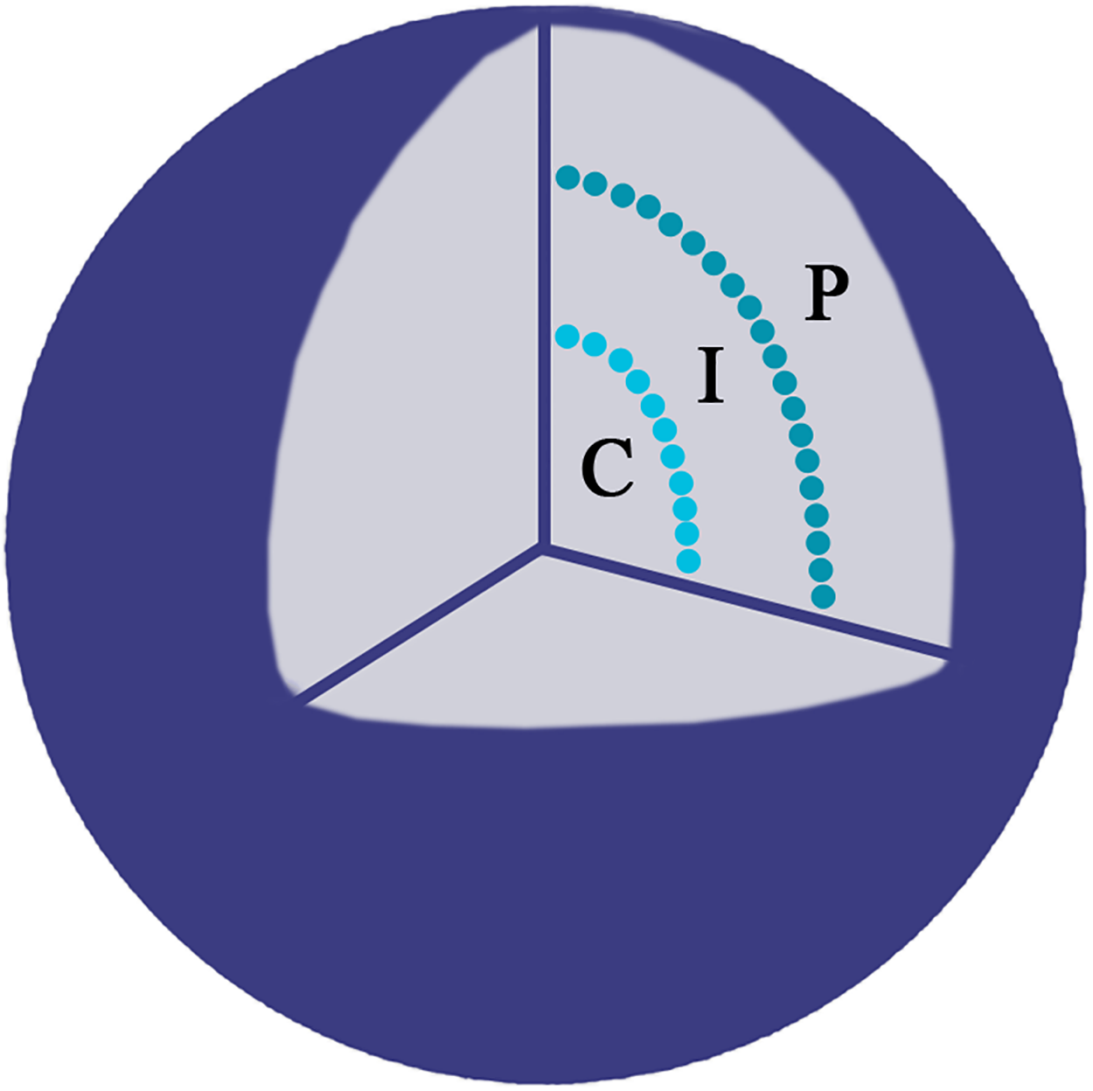
A schematic representation of the nucleus. The radial position of the studied genes was evaluated as a distance from the FISH signal to the nuclear border, divided by the distance from the FISH signal to the nucleus centre. Three locations were considered central (C), intermediate (I) and peripheral (P).

To determine the radial position of the studied genes and the CT’s the ratio of the measured distance was calculated (*R* = distance from the FISH signal to the nuclear border divided by the distance from the FISH signal to the nucleus centre) as described by Kociucka et al. [36] with modifications. The value *R ≥* 2 was considered as nuclear centre (C), 2 > *R* ≥ 0.5 was considered as an intermediate (I) position and *R* < 0.5 as nuclear periphery (P) as indicated in Fig 1.

The location of a gene within its CT and the shape of the CT were evaluated with NEMO graphical interface. Three possible gene locations within the CTs were observed: outside of the CT, on the CT surface and inside of the CT. The shape of the CT was determined according to Seghal et al. [37]. Three shape variants were observed: (1) regular—compact, ellipsoid; (2) intermediate—oval with irregular edge, with at least one protrusion; (3) irregular with highly convoluted borders and numerous protrusions, often shaped like a ribbon.

### Quantitative gene expression analysis (Q-PCR)

RNA extraction and reverse transcription were performed as previously described by Madeja et al. [19]. RNA extraction was performed using High Pure miRNA Isolation Kit (Roche Diagnostics). After RNA precipitation (Pellet Paint NF Co-Precipitant; Novagen, USA) cDNA synthesis was carried out with Transcriptor High Fidelity cDNA Synthesis Kit (Roche Diagnostics). The standard curve method and the real time absolute quantification methods were used to determine the number of copies of the analysed genes (*CDX2*, *OCT4*, *NANOG*). The concentration of extracted DNA was verified by Nanodrop (Thermo Scientific Inc.). Relying on the DNA concentration, 10-fold serial dilutions were prepared to obtain the standard curves for each gene. These DNA standards were used as reference templates in each Q-PCR reaction. The reactions were performed on Light Cycler 2.0 (Roche Diagnostics). The data were normalized using the geometric mean of the expression levels of the endogenous genes *GAPDH* and *YWHAZ*, which have been previously described as appropriate reference genes for bovine preimplantation embryos [38, 39]. For each developmental stage 4 biological replicates were performed. Each reaction was performed in duplicates. The primer pairs (Table 2) were the same as used by Madeja et al. [19] the reference genes were based on Goossens et al. [40]. The reactions were carried out in 10 μl capillaries (Roche Diagnostics) and the PCR mix comprised of 1 μl of Light Cycler Fast Start DNA master SYBR Green (Roche Diagnostics), 5mM MgCl2 (Roche Diagnostics), 0.5 μM of primers and 2 μl of cDNA. Q-PCR reaction conditions included: initial polymerase activation at 95°C for 10 min, 45–50 cycles of denaturation at 95°C for 10s, primer specific annealing (listed Table 2) for 10s followed by final elongation at 72°C for 10s. Product specificity was confirmed by melting analysis.

**Table 2:** Primer pairs and annealing conditions used for gene expression analyses.

Name	Sense primer	Antisense primer	Product size	Annealing temperature
*CDX2*	CTTTCCTCCGGATGGTGATA	AGCCAAGTGAAAACCAGGAC	113 bp	63°C
*OCT4*	GTTTTGAGGCTTTGCAGCTC	CTCCAGGTTGCCTCTCACTC	185 bp	63°C
*NANOG*	AAACAACTGGCCGAGGAATA	AGGAGTGGTTGCTCCAAGAC	194 bp	63°C
*GAPDH*	TTCAACGGCACAGTCAAGG	ACATACTCAGCACCAGCATCAC	119 bp	63°C
*YWHAZ*	GCATCCCACAGACTATTTCC	GCAAAGACAATGACAGACCA	120 bp	63°C

### Immunofluorescent protein labelling

The antibodies, the procedure and the appropriate control experiments were done according to our previously optimised protocol [19]. After collection, the embryos were briefly washed in PBS (pH 7.3) and for embryonic stages other than hatched blastocysts, *zona pellucida* was removed prior to fixation, by brief incubation in acidic Tyrode’s solution with 0.1N HCl. Embryos were fixed in 4% PFA in PBS with 0.1% Triton X-100 and 0.1% Tween-20 (pH 8.0) for 15 min in 39°C. The staining procedure included permeabilisation with 0.55% Triton X-100 in PBS for 20 min followed by blocking of nonspecific antibody binding sites with 10% foetal calf serum (FCS) in PBS (60 min at room temperature, RT). Next, the embryos were washed 3x (5min at RT) in 0.1% Triton X-100 in PBS (PBX) and subjected to an o/n (+4°C) with the primary antibody (ABI) diluted 1:50 in the blocking buffer. ABI was removed by 3 washes in PBX followed by 40 min blocking at RT and 1.5h incubation (at RT) with the secondary antibody (ABII) at 1:200 dilution. Finally the ABII was removed by 3 washes in PBX and mounted on 18-well μ-slides (tissue culture treated, Ibidi Gmbh) in 40μl drop containing antifade solution with DAPI (Vectashield mounting medium, Vector Laboratories). The slides were stored at +4°C.

To verify the signal specificity we have used various sets of primary and secondary ABs. The ABI set included: anti-CDX2 mouse monoclonal AB (Abcam, UK, ab15258); anti-CDX2 rabbit polyclonal AB (ab88129); anti-OCT4 rabbit polyclonal AB (Abcam, ab18976) or goat polyclonal AB (Abcam, ab27985); anti-NANOG rabbit polyclonal AB (PeproTech, UK, 500-P236). The ABII set consisted of Santa Cruz Biotechnology (USA) antibodies: donkey anti-rabbit IgG-Rhodamine conjugated AB (sc-2095), goat anti-mouse IgG-Rhodamine conjugated AB (sc-2092), goat anti-mouse IgG-FITC conjugated AB (sc-2010) goat anti-rabbit IgG-Rhodamine conjugated (sc-2091), mouse anti-goat IgG-FITC conjugated (sc-2862), mouse anti-goat IgG-Rhodamine conjugated (sc-2490). Examples of negative control experiments are provided in S1 Fig. Fluorescent signals were visualized using Zeiss Axiovert 200M laser scanning confocal microscope.

### Statistical analysis

Statistical analyses were performed with IBM SPSS Statistics 24.0 Software. All variables were tested for normality using Kolmogorov-Smirnov test. The differences in mRNA expression level between the developmental stages were analysed with Kruskal-Wallis and Mann Whitney test. The frequencies of CT and locus localization patterns and CT shapes were analysed with chi square and Z tests. A two-sided P value of <0.05 was considered statistically significant.

## Results

### Radial position of CTs containing lineage specific genes during early development is related to chromosome size

**Fig 2:**
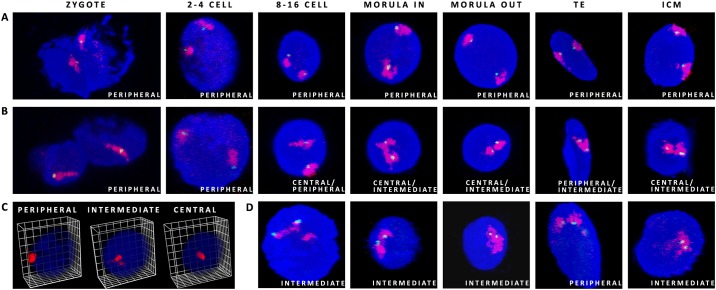
Possible CT positions within the nuclei of bovine embryos. (A) CT12 (red) with the *CDX2* locus (green) present an example of peripheral location, (B) CT23 (red) with the *OCT4* locus (green) indicate central to intermediate location from 8–16 cell stage onwards. (C) presents possible radial distribution of CTs within the 3D nuclear space, (D) CT5 (red) with the *NANOG* locus (green) show an example of intermediate location in 8–16, morula and ICM. Confocal sections were taken every 0.5μm (zygotes to 8–16 cell stage) and every 0.4μm for morulae and blastocysts. DAPI stains the chromatin.

**Fig 3:**
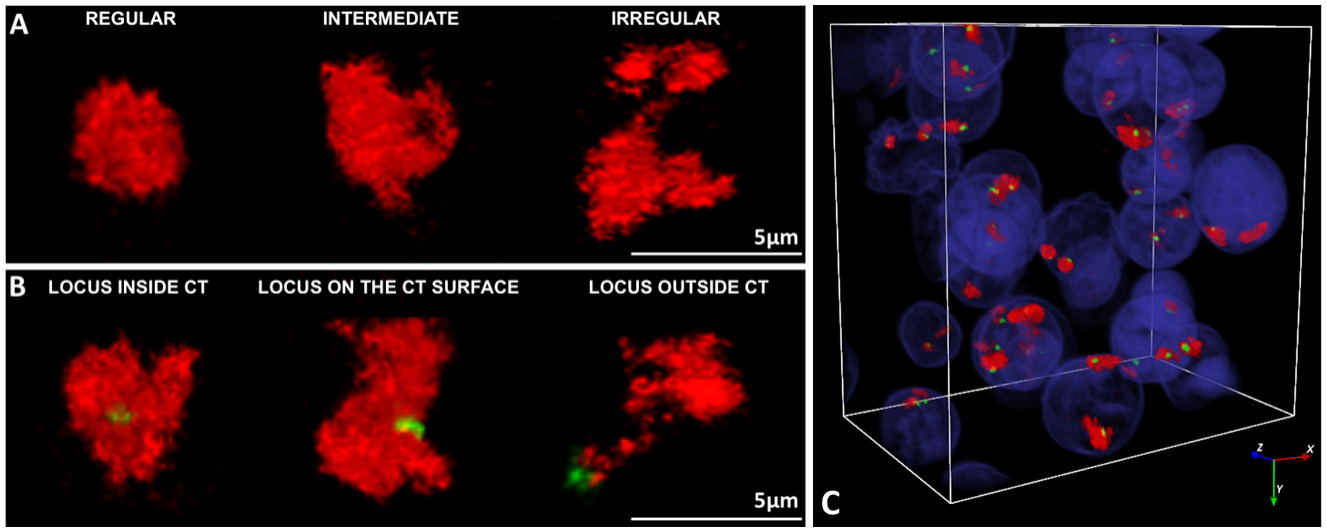
**Chromosome territory shape variants** (A) **and observed locus positioning** (B) **within the CT**. (C) presents partial 3D reconstruction of a morula stage embryo from images taken for CT23 (red) and the *OCT4* locus (green). Confocal sections were taken every 0.4μm. DAPI labels chromatin.

The radial distribution of chosen CTs in the nuclei was followed from the zygote to the blastocyst stage (9dpi). A special attention was put on crucial developmental stages: 8–16 and morula (where the inner and the outer cell nuclei were evaluated separately). First we have analysed large (91.2Mbps) gene poor BTA12 carrying the total number of 396 genes (including the *CDX2* locus), with an average density of 4.3genes per 1Mbp and much smaller BTA23 (52.5Mbp) containing up to 762 genes. BTA23 also locates the *OCT4* gene locus and overall has three times more genes per 1Mbp (14.5) than BTA12, being one of five chromosomes with the highest gene density in bovine genome. Because the predominant location of these 2 CTs was peripheral, with a noted shift towards the intermediate position around the time of EGA for CT23, we have also decided to include BTA5 containing the *NANOG* locus (120Mbps). It falls within the top 5 of bovine autosomes carrying the highest number of genes (about 1300), with a moderate gene density of about 10.61Mbp. It has been hypothesised that CTs of these chromosomes could behave differently, depending on their gene density and/or on corresponding transcriptional activity. The examples of the observed CT shape variants and locus positions are presented in Fig 2 and CT distribution patterns and a 3D reconstruction of a morula stage embryo are documented in Fig 3. Complete 3D reconstructions are provided in S1–S4 Movies.

**Fig 4:**
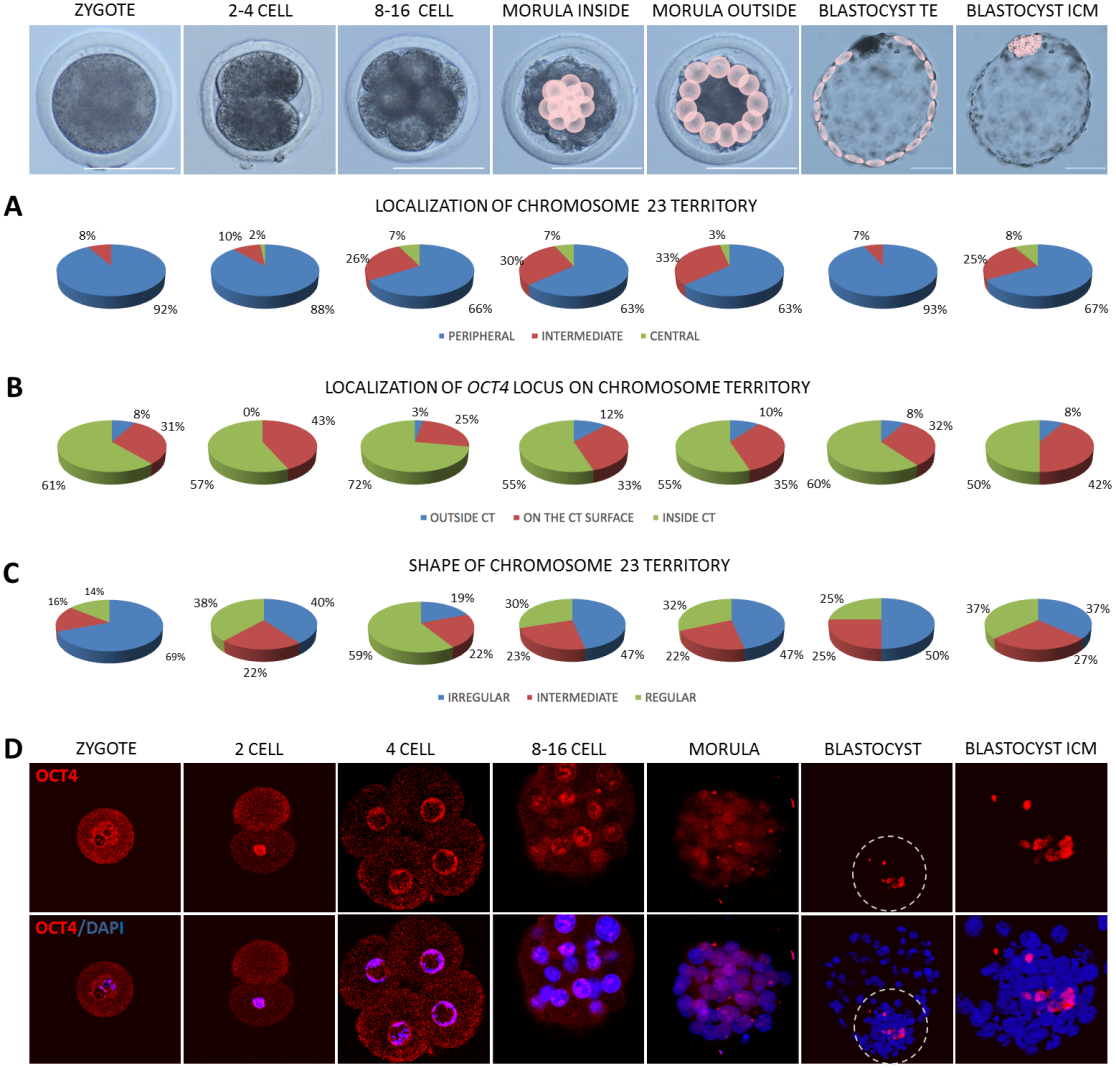
Summary of data obtained for CT23 and the *OCT4* locus. Top panel presents the analysed developmental stages, pink circles point to cells for which analyses were carried out at morula and blastocyst stages. (A) summarises the contribution of peripheral, intermediate and central location of CT23 within the analysed nuclei, (B) presents localisation of the *OCT4* locus in relation to its CT, (C) indicates changes in CT23 shape related to embryonic stage. (D) presents OCT4 localisation (red immunofluorescence) in bovine embryos. The encircled region indicates the ICM, highlighted in the far right images. Chromatin was visualised by DAPI, confocal sections were taken every 4μm. Scale bar:100μm.

**Fig 5:**
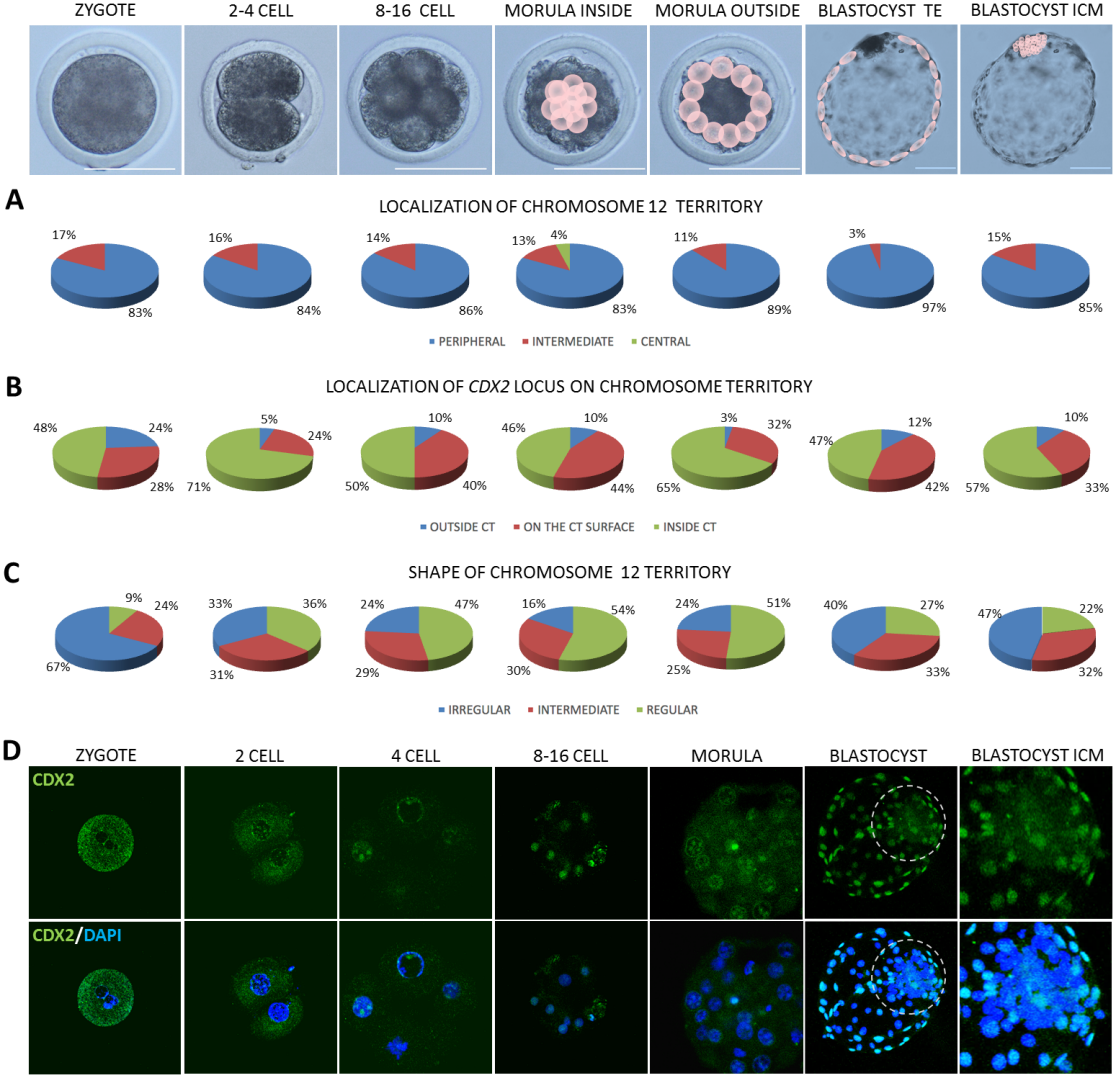
Summary of data obtained for CT12 and the *CDX2* locus. Top panel presents the analysed developmental stages, pink circles point to cells for which analyses were carried out morula and blastocyst stages. (A) summarises the contribution of peripheral, intermediate and central location of CT12 within the analysed nuclei, (B) shows the localisation of the *CDX2* locus in relation to its CT, (C) indicates the changes in CT12 shape related to embryonic stage. (D) presents immunofluorescent localisation of CDX2 (green) in bovine embryos. The encircled region indicates the ICM, highlighted in the far right images. Chromatin was visualised by DAPI, confocal sections were taken every 4μm. Scale bar: 100μm.

**Fig 6:**
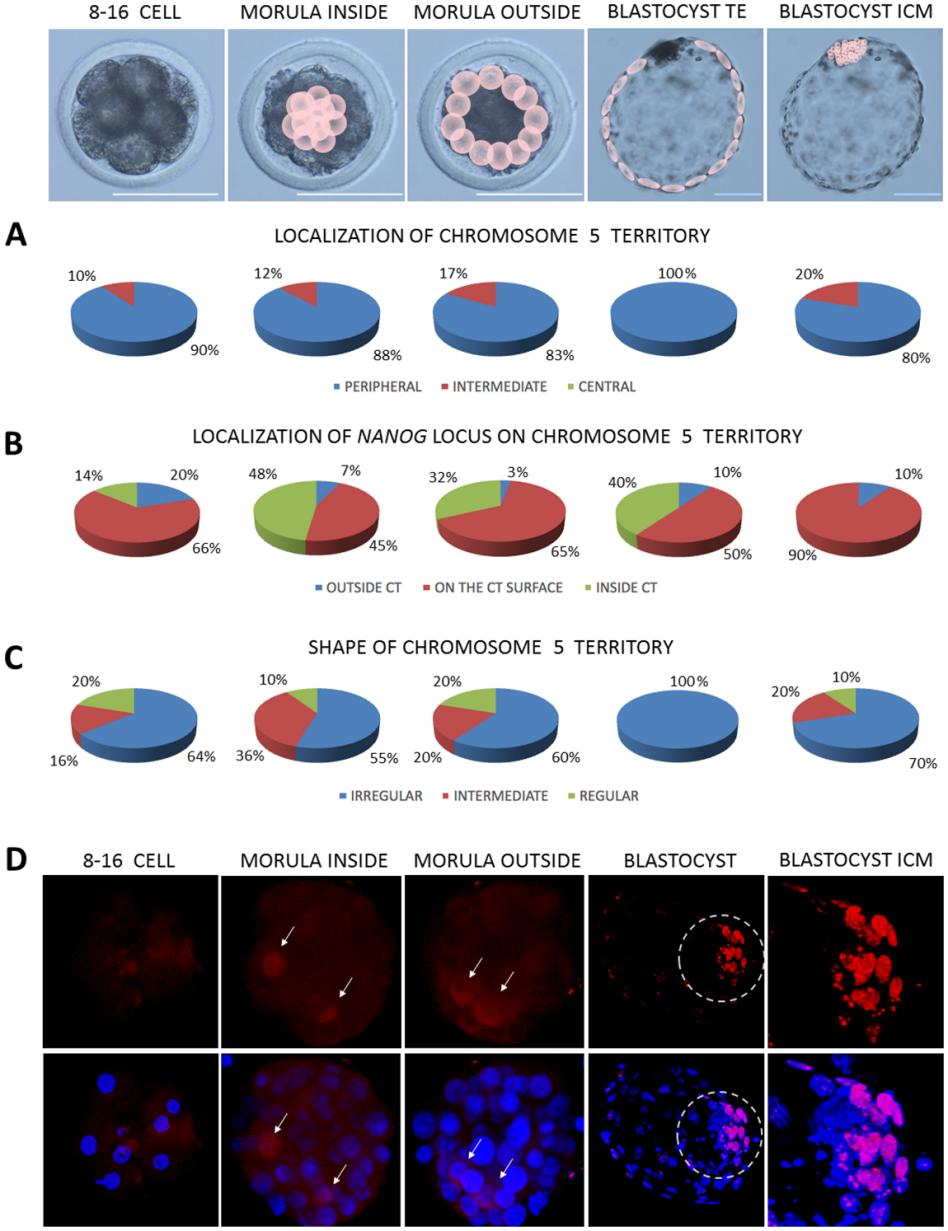
Summary of data obtained for CT5 and the *NANOG* locus. Top panel presents the analysed developmental stages, pink circles point to cells for which analyses were carried out at morula and blastocyst stages. (A) summarises the contribution of peripheral, intermediate and central location of CT5 within the analysed nuclei, (B) shows the localisation of the *NANOG* locus in relation to its CT, (C) indicates the changes in CT5 shape related to embryonic stage. (D) presents NANOG localisation (red immunofluorescence) in bovine embryos. The encircled region indicates the ICM, highlighted in the far right images. Arrows indicate NANOG positive cells in morula inner and outer cells. Chromatin was visualised by DAPI, confocal sections were taken every 4μm. Scale bar: 100μm.

As already stated, CTs mostly localised peripherally (P) despite of the analysed developmental stages (Figs 4A, 5A and 6A). However, at 2–4 cell stage central (C) location of CT23 appeared in 2% of the analysed cells, intermediate (I) in 10% and the remaining 88% was peripheral. CT23 substantially changed its location to I and C starting from 8–16 cell stage (P<0.01, Fig 4A). At the same developmental stages large CT12 remained relatively stable in its P position with the highest frequency in TE (97%) compared to other stages (P<0.05). CT12 did not exhibit C location, except for the 4% of the internal cells of the morula stage embryo. The I location of CT12 accounts for several percent, with the highest score in zygotes (17%) and 2–4 blastomere embryos (16%) followed by a slight decrease in the subsequent developmental stages (Fig 5A). In contrast, BTA23 (smaller but rich in genes) at 8–16 cell and morula stages 2 to 3 times more frequently displayed the I location (26%–33%) than CT12 (14%-11%). The largest and gene rich CT5 at 8–16 cell stage only in 10% displayed the I location, the remaining 90% was peripheral (Fig 6A). In the following developmental stage, this overall proportion did not change, with 12% I and 88% P in the interior morula cells and 17% I and 83% in the outer morula cells.

The major burst of bovine EGA arise at 8–16 cell stage, therefore notable changes in the location of CT23 may reflect high requirement for transcriptional activity, a pattern which was preserved beyond the morula stage. At the blastocyst stage, where the embryonic lineages are separated into the ICM and the TE, the investigated CTs predominantly located at the nuclear periphery. Significant differences were noted for the two lineages in CT12 distribution which in 97% located peripherally in the TE and in 85% in the ICM (P<0.05). CT23 showed a pronounced difference in the frequency of I and C location (P<0.05) between the TE (7%) and the ICM (33%) suggesting plausible accessibility to the transcription machinery in the ICM and silencing in the TE. In case of CT23 every third cell of the ICM was characterised by C (8%) or I location (25%) being twice as likely as in the case of gene deficient CT12 (Figs 4A and 5A) Moreover, no C location of CT23 was observed in the TE cells. Interestingly, the largest CT5 solely located peripherally in the TE cells nuclei (100%, P<0.01), whereas in the ICM 20% of its location was I and 80% P (Fig 6A).

### Gene locus position within its CT is related to stage specific embryonic lineage differentiation

Loci location of the investigated genes in relation to their CTs revealed a variable pattern of positioning: within (inside) or outside of the CT (Figs 4B, 5B and 6B). According to the theories transcriptionally active regions (genes) may temporarily loop out of the CT becoming accessible to the molecular machinery at a precise time point needed. In zygotes in 61% *OCT4* located inside the CT, only in 8% is positioned outside of CT23. At 2–4 cell stage which precedes the EGA the incidence of peripheral location increased to 43%. At the time of EGA in 3% of cases *OCT4* locus moved away from the CT, 25% of signals was detected on the surface and 72% remained inside. Further shift outside CT23 was visible in morula stage embryos (12% inner cells and 10% outer cells, P<0.01). Similar frequencies were noted for the *OCT4* locus in blastocysts. Both in TE and ICM there was an 8% incidence of *OCT4* locus locating outside of the CT. Similarly, the *CDX2* locus changes its position during preimplantation development. In zygotes in 48% of cases it located inside CT12, in 28% on the surface and in 24% outside (Fig 5B). In 2–4 cell stage embryos, similarly to *OCT4*, *CDX2* locus changes its position towards the centre of the CT (71%) and only in 5% remains outside of CT12. This observation is significantly different (P<0.01) from morula inner cells and TE.At the time of EGA (8–16 cell stage) the incidence of *CDX2* locus being outside of the CT increased to 10%, a pattern which is preserved within the inner population of cells of the morula stage embryo. The outside cells presented mainly the inner location of the *CDX2* locus in relation to CT12 and only 3% remained outside or the CT. These changes could reflect the dynamic alternations in expression and regulation of developmentally important factors, especially that it was proven that up to certain stage, the embryonic cells may temporarily change their gene expression patterns. Following the notion of the inside/outside theory of lineage segregation, the *CDX2* locus was found predominantly on the surface of CT12 (42%) and outside of the CT (12%) in the trophoblast cells, suggesting its availability for transcription. This observation supports the important role of CDX2 in TE lineage maintenance. Concomitantly in the ICM the majority of the analysed CTs displayed internal location of *CDX2* (57%).

On the contrary to *OCT4* (predominant inner location) *NANOG* locus was mostly detected on the surface of CT5 (Fig 6B). In 8–16 cell stage embryos the outside/surface location accounted for 86% of the cell nuclei analysed. Within the inner population of cells of the morula stage embryo the proportion has changed to a 52% incidence of *NANOG* locus being positioned on the surface (45%) and outside (7%). In 48% of the inner morula cell’s nuclei *NANOG* was located inside the CT. In the outside cells of the morula in 32% (P<0.01) of observations the locus was still placed internally, however the incidence of the outside and surface location combined increased to 67%. This pattern was somewhat preserved in the TE cells, where 10% *NANOG* loci were positioned outside of the CT and 60% were on the surface. Concomitant with our knowledge of NANOG’s function at the blastocyst stage, in the ICM only surface (90%) and outside (10%) location was observed suggesting a correlation with its high transcriptional activity that has been previously reported by our group [19]. These frequencies were among the highest observed the investigated developmental stages (P<0.01).

### Chromosome territories change their shape across developmental stages

Higher transcriptional activity is attributed to the irregular shape of the CT. Our results show that changes in shape of CT5, CT12 and CT23 are related to the developmental stage (Figs 4C, 5C and 6C). The incidence of irregular shape decreased from zygote to 8–16 cell stage for CT23 (P<0.05) and up to morula stage for CT12 (P<0.05). This was followed by an increase observed in the blastocyst stage. The highest frequency of irregular shape was noticed in zygotes for both CT12 (67%) and CT23 (69%) while the lowest in 8–16 cell stage embryos (CT12-24%, CT23-19%, P<0.01) and morulae (CT12-20%), what coincides with increasing transcriptional activity at the time EGA. Interestingly CT23 showed much higher probability of acquiring the irregular shape in morula inside and outside cells (47%) as compared to CT12 (16% and 24% respectively, P<0.05). In blastocysts CT12 changed its shape to irregular in 47% of cell nuclei analysed for the ICM and in 40% of TE cells. In contrast CT23 shape was in 50% irregular in the TE and only 37% in the ICM. CT5 showed the highest frequency of irregular shape in TE (100%) compared to ICM (70%) and other stages (64% in 8–16 cell, 55% and 60% in inside and outside morula cells; P<0.01). Interestingly CT5 showed the highest irregular shape in blastocyst stage embryos compared to CT23 and CT12. Intermediate and regular CT5 were observed with similar frequencies in all analysed stages despite TE cells, were only irregular shape was present.

The proportion of irregular shape of CT5 is somewhat reversed to the observations made for the other two chromosome territories (Fig 6C). The incidence of irregular shape was predominant at all of the analysed developmental stages (from 8–16 cell up to the blastocyst stage). At the time of EGA irregular shape was attributed to 64% of the analysed cell nuclei, 20% was regular. This pattern was mostly preserved at the morula stage, where in case of the internal cells 55% retained the irregular shape (with 10% regular). In the outside cells in 60% of the analysed cases CT5 shape was irregular, in 20% both intermediate and regular. The striking difference was noted for the blastocysts, where in the nuclei of the TE cells all of the analysed CTs presented the irregular shape (100%). In the ICM however, the proportion was 70% irregular, 20% intermediate and 10% regular. This observation provides interesting cues on stage specific and pluripotency related changes in 3D chromatin organisation within the nucleus.

### Changes in transcript abundance correlate with protein distribution patterns

**Fig 7:**
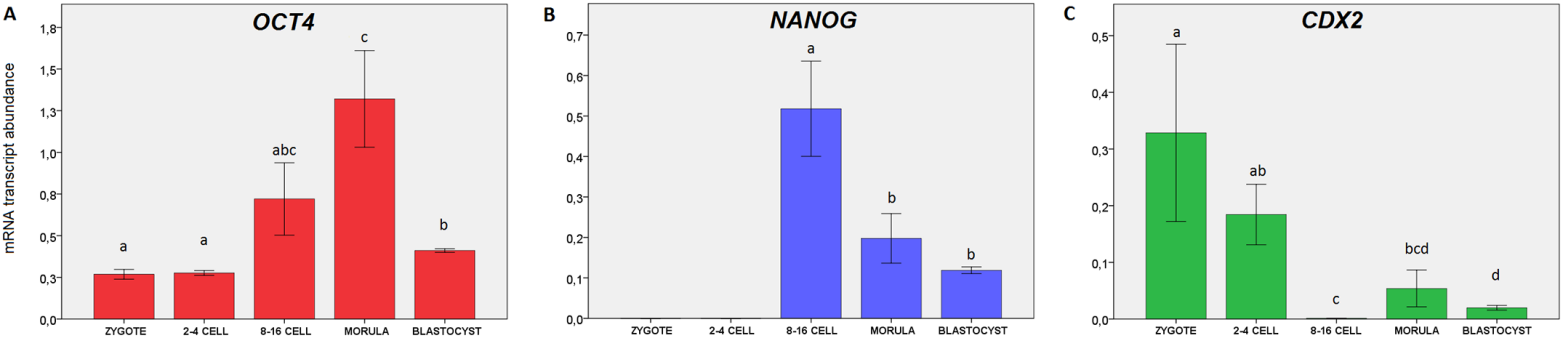
Gene expression profiles of lineage specific markers during bovine preimplantation development. (A) indicates the relative changes in transcript abundance for *OCT4*, (B) for *NANOG* and (C) for *CDX2*. The bars (± SEM) labelled with different letters indicate stages which significantly differ in transcript abundance (P≤0.05).

As expected, *OCT4* transcripts were detected at all of the studied stages, however the mRNA abundance showed stage specific dynamics (Fig 7A). The lowest expression levels were noted for zygotes and 2–4 cell stage embryos, suggesting the maternal origin of transcripts. Maternal OCT4 is accepted to play a role in initiating totipotency and acquisition of oocyte developmental competence [41, 42]. To supplement this data we have also detected OCT4 protein and transcripts in bovine GV and MII oocytes (S2 Fig). Corresponding to the time of bovine genome activation, starting from the 8–16 cell stage *OCT4* mRNA level steadily rose reaching the peak at the morula stage. These differences were statistically significant for morulae, zygotes (P≤0.05) and 2–4 cell embryos (P≤0.05). At the blastocyst stage *OCT4* level dropped, but still remained significantly higher than in zygotes and 2–4 cell embryos (P≤0.05) reflecting embryonic lineage specification. This data remains in agreement with the OCT4 protein distribution pattern (Fig 4D). In zygotes OCT4 was located in the cytoplasm, condensing around the pronuclei. At the 2-cell stage the cytoplasmic location was still visible, however the majority of the analysed embryos (6/8) displayed strong nuclear signal. At the 4-cell stage OCT4 was predominantly nuclear (all cells). Concurrent with the subsequent cell divisions (starting from 8–16 cell stage) OCT4 became restricted to the nuclei. At the time of EGA developmentally important transcription factors ignite lineage specification. Prior to the ICM formation there is an increasing need for the expression of pluripotency related factors (reflected by an increase of the specific mRNA abundance at the morula stage). Thus in morulae we have observed strong nuclear signals for OCT4, which were predominantly visible in the inner group of cells (Fig 4D, magnified ICM area) already leaving distinct outside cells devoid of the signal. Finally at the blastocyst stage OCT4 positive cells specifically populated the ICM.

In contrast to *OCT4*, *NANOG* gene expression was detected at a high level starting from the 8–16 cell stage (transcripts were not detected in zygotes and 2–4 cell stage embryos), suggesting *de novo* synthesis (Fig 7B). At the morula sage the mRNA level dropped (P≤0.05) and was steadily maintained in the blastocyst. Concomitant with this data NANOG protein was first detected in a small proportion of cells of the morula stage embryo–cytoplasmic in the outside cells and nuclear in the inner cells (Fig 6D). At the blastocyst stage (9dpi) NANOG signal was observed only within the ICM group of cells (Fig 6D magnified area).

*CDX2* mRNA was detected at all of the analysed preimplantation stages. The mRNA level declined from zygote to 8–16 cell stage and increased again at the morula and blastocyst stage (Fig 7C). This corresponds to the observations made for the rhesus macaque where *CDX2* expression was markedly upregulated in pronuclear stage zygotes and drastically diminished in cleavage stage embryos [25]. Similarly, our study shows significantly higher *CDX2* transcript abundance in zygotes than in 8–16 cell stage, morulae and blastocysts (P≤0.05) suggesting maternal mRNA degradation/silencing. The lowest mRNA level was noted at the time of bovine EGA (8–16 cell stage) representing a typical embryonic gene expression pattern. These differences were indicative for 2–4 cell stage and blastocysts (P≤0.05). At the protein level CDX2 distribution pattern complements the mRNA data. In zygotes and in early embryos (2-cell) some CDX2 signals are detected within the cytoplasm, but at the later stage embryos CDX2 located within the cell nuclei, a pattern which is conserved until the blastocyst stage (Fig 5D). However, at the time of bovine blastocyst formation (starting from 7dpi) CDX2 segregation to the trophoblast lineage becomes apparent. Finally at the late blastocyst stage (hatched blastocyst, 9dpi) only the trophoblast cells retain CDX2 positive signals (Fig 5D).

## Discussion

Our understanding of processes linking chromatin arrangement within the 3D nuclear space with transcriptional activity of developmentally important factors in mammalian embryos is still limited. Studies indicate that nuclear architecture is correlated with and underlines gene expression, but phenotypic consequences of altering the 3D genome organisation particularly in relation to early development (and pluripotency) are not well understood. It is anticipated that chromatin compaction can influence transcriptional activity by regulating accessibility to transcription factors and DNA interactions. In differentiated cells, the location of the chromosome within the nucleus is thought to control gene activity, with nuclear periphery associated with transcriptionally repressed chromatin [rev by [43]]. Ahmed et al. [31] showed that chromatin in mouse zygote pronuclei was evenly distributed with a minimal degree of concentration which occurred at the surface of nucleolar precursor bodies and at the nuclear envelope. At this stage, the distinct structural compartmentalisation of chromatin may not be required, as the embryonic genome is uniformly under-expressed. Low level of transcription does occur, but the majority of transcripts do not produce mature mRNA [44]. The major burst of mouse EGA occur at the 2-cell stage, prior to which chromatin becomes structured and compacted domains appear. A 3D-DNA-FISH study of bovine embryos revealed that prior to the EGA, gene density of a chromosome did not correlate with its radial distribution. However, after genome activation gene poor chromosomes tend to occupy more peripheral territories [2]. In cattle the process of EGA is gradual and may be divided into early gene activation (zygotes, 2-cell embryos) and mid genome activation (2–5 cell stage), accompanied by *de novo* RNA synthesis. The 8–16 cell stage represents the major burst of gene activity [45, 46].

To test the hypothesis that differentiation should be marked by global changes in chromosome positioning within the nucleus, we have followed the 3D architecture of bovine interphase chromosomes (BTA12,BTA23 and BTA5). In principle, the establishment of pluripotency should require chromatin changes, that allow stable expression of the key transcription factors. The ICM specific *OCT4* locus is placed on gene rich small BTA23 and the TE specific CDX2 on large gene poor BTA12. Prior to the EGA both CTs predominantly display P/I location, being in agreement with the above theories. At this point transcripts and proteins are mainly of maternal origin, thus no need for gene expression related CT displacement. Knowing the gradual process of bovine genome activation, at 2–4 cell stage we have observed a slight shift of CT23 towards the centre of the nucleus. At 8–16 cell stage the proportion of I/C location further increased, but still the majority of the analysed embryonic cells displayed peripheral location. At this stage most of maternally derived transcripts should be silenced or degraded. At the molecular level we have observed low level of *OCT4* transcripts in zygotes and 2–4 cell embryos. A significant increase in *OCT4* mRNA level around the time of EGA was accompanied by an increase in the incidence of I/C location of CT23. This pattern was maintained at the morula stage where the inner cells displayed slightly higher proportion of central location than the outer cells. This observation coincides with the timing of cellular specification in mammalian embryos and is related to the rising levels of lineage specific factors such as OCT4, NANOG and CDX2. Yet, the molecular processes underlying the first cell fate decisions begin much earlier, as lineage specific markers are variably co-expressed in individual cells prior to the EGA. In stages preceding the ICM/TE segregation OCT4 positive signals were detected in all cell nuclei. Only in zygotes the localisation was cytoplasmic/sub-nuclear, reflecting a pattern observed in oocytes. At the morula stage OCT4 positive nuclei were observed predominantly within the central group of cells. The specific marker gene expression becomes mutually exclusive as cells become lineage committed [47]. This is supported by our observations as *OCT4* transcripts maintain high expression levels at morula and blastocyst stages. Furthermore, our previous study indicate that OCT4 protein exclusively locates in the ICM of bovine blastocysts (9dpi) and is accompanied by a 4-fold higher transcript expression than in the TE [19]. This observation supports the hypothesis linking CT activity with its position within the nucleus. Bovine TE cells show no central location of CT23. At this stage OCT4 expression is overridden by mutual repressive effects of CDX2 leading to the establishment of a CDX2-positive/OCT4-negative outside compartment [48]. According to the theories, BTA23 may be laminin bound at the periphery and less accessible to transcription machinery. Peripheral localisation at the nuclear lamina has been observed for silent tissue specific promoters, while active promoters present likelihood of being internal. The loss of developmental plasticity correlates with compaction of genomic chromatin, not only into domains associated with H3K27 methylation [49], but also with the nuclear lamina. Studies indicate that lamina-genome interactions may be involved in the control of gene expression programs during lineage commitment and terminal differentiation [50].

The *CDX2* gene expression profile follows slightly different pattern than the *OCT4*. A drastic reduction in transcript content was noted from zygote to 8–16 cell stage. Relatively high expression in zygotes corresponds to data presented by Wu et al. [51] who detected high levels of *CDX2* transcripts in MII oocytes, which declined at the 8 cell stage, and similarly to our data rose in the subsequent developmental stages. Thus, after the drastic degradation of maternal mRNA, *CDX2* gene needs to become accessible to the transcriptional machinery. The results of our observations indicate that CT12 does not substantially relocate at the analysed stages and remains mainly peripheral. Nuclear periphery does not necessarily impose transcriptional repression. Most chromosomes do not change their radial position during differentiation in human ESC, which generally have gene density related radial organisation of chromosomes, just like in differentiating cells [52]. This phenomenon seems to be universal, as both in mice and *C*. *elegans* compact and transcriptionally silent heterochromatin appears progressively as cells differentiate and lose their capacity to be reprogrammed to another cell fate [49]. In embryonic cells heterochromatic high copy number repeats are located at the nuclear rim, while low copy repeats do not show preferential localisation. At the onset of differentiation, activation of promoters is able to overcome the anchoring to the nuclear periphery, so CTs containing developmentally important transcription factors do not need to substantially relocate. Adequately studies on human ESC revealed that large chromosome 6 (which contains the *OCT4* locus) does not change its overall nuclear position during differentiation. Instead, the locus relocates to a position outside its CT [52]. Therefore, the observed changes in the *CDX2* gene position within bovine CT12 may potentially be associated with activation of transcription. In 8–16 cell stage embryos peripheral and surface location of the *CDX2* locus accounts for 50% of the analysed cells. In morula stage we have observed a difference in the proportion of outer/surface location between the inner (54%) and the outer (35%) cells, which considering the theories of lineage formation may be surprising. However, this could be explained by the dynamic molecular changes happening at this stage. In mouse embryos an overlapping expression of lineage specific factors was observed up to the morula stage irrespective of cell’s position within the embryo [47]. In bovine, just like in mouse embryos, a clear TE lineage segregation of CDX2 becomes visible at the late blastocyst stage [19]. Studies of *NANOG* locus distribution within its CT complement the above observations. Large chromosomes may preferentially occupy peripheral regions of the nucleus, yet remain transcriptionally active due to positional remodelling of chromatin. In our observations CT5 did not substantially change its peripheral localisation, but at the same time *NANOG* locus was predominantly positioned on the surface or outside of CT5. At the molecular level *NANOG* gene transcripts were first detected at 8–16 cell stage, with first immunofluorescent signals noted at the morula stage. Importantly in the ICM cells, where *NANOG* gene expression is essential for EPI formation its locus in 90% was noted on the surface—no internal location was observed. Additionally the whole CT5 slightly shifted towards the centre of the nucleus, in contrast in the trophoblast cells where its location was solely peripheral, with 40% *NANOG* loci detected inside CT5. This coincides with protein localisation, which is strictly ICM specific.

TE lineage establishment is the first specification event in mammalian preimplantation development. In the mouse this process is achieved via a feedback loop regulated by ELF5 which results in the mutual suppression between CDX2, OCT3/4 and NANOG complex. Functional studies showed that CDX2 blocks OCT3/4 activity in the TE committed cells. As a result of this interaction, both of these factors may change their localisation to transcriptionally inactive regions of the nucleus. It was shown that in the presence of CDX2, OCT3/4 occupied heterochromatic regions of the nuclei [10]. In cow this interaction is not as evident as bovine *OCT4* locus does not contain the *cis*-acting regulatory region necessary for extinguishing its transcription by CDX2 [53]. However, in our previous study on the effects of β-catenin signalling on bovine preimplantation development, we have showed that the maintenance of WNT signalling during preimplantation development results in ubiquitous expression of OCT4 and a decrease in the *CDX2* transcription and protein distribution (with some embryos devoid of CDX2), suggesting a level of interaction between these factors [54]. Our observation may be in part supported by the studies of early porcine embryos where CDX2 repressed OCT4 at the post translational level by inducing proteasomal degradation of OCT4, and not by repressing its transcription and translation [55].

Morphological evaluation of CTs chosen for this study suggest that the observed changes in CT shape may also correlate with transcriptional activity of the analysed genes. Generally gene-rich domains are less condensed and of more irregular shape than gene-poor domains and open chromatin can create the optimal environment for transcriptional activity [56]. However, high gene density is not always equivalent to gene activity, as inactive genes can also be present in open chromatin domains [57]. Although the transcriptional activity of whole CT’s has not been studied here, it was previously shown that in bovine embryos the overall number of activated genes increased from the 8–16 cell stage onwards [58]. A complex study of 12 human chromosomes differing in size and gene density revealed a correlation between gene density and CT shape [37]. The incidence of the irregular shape of CTs investigated by us followed the changing dynamics of early embryonic development. Gene-rich CTs 5 and 23 and gene-poor CT12 exhibited a similar pattern of irregular shape acquisition which is generally attributed to active chromatin. Data supporting this observation comes from studies comparing the shape of active and inactive X chromosomes in human amniotic fluid cells [57]. We suppose that the CT shape may be influenced by the overall gene activity on a given chromosome.

**Fig 8:**
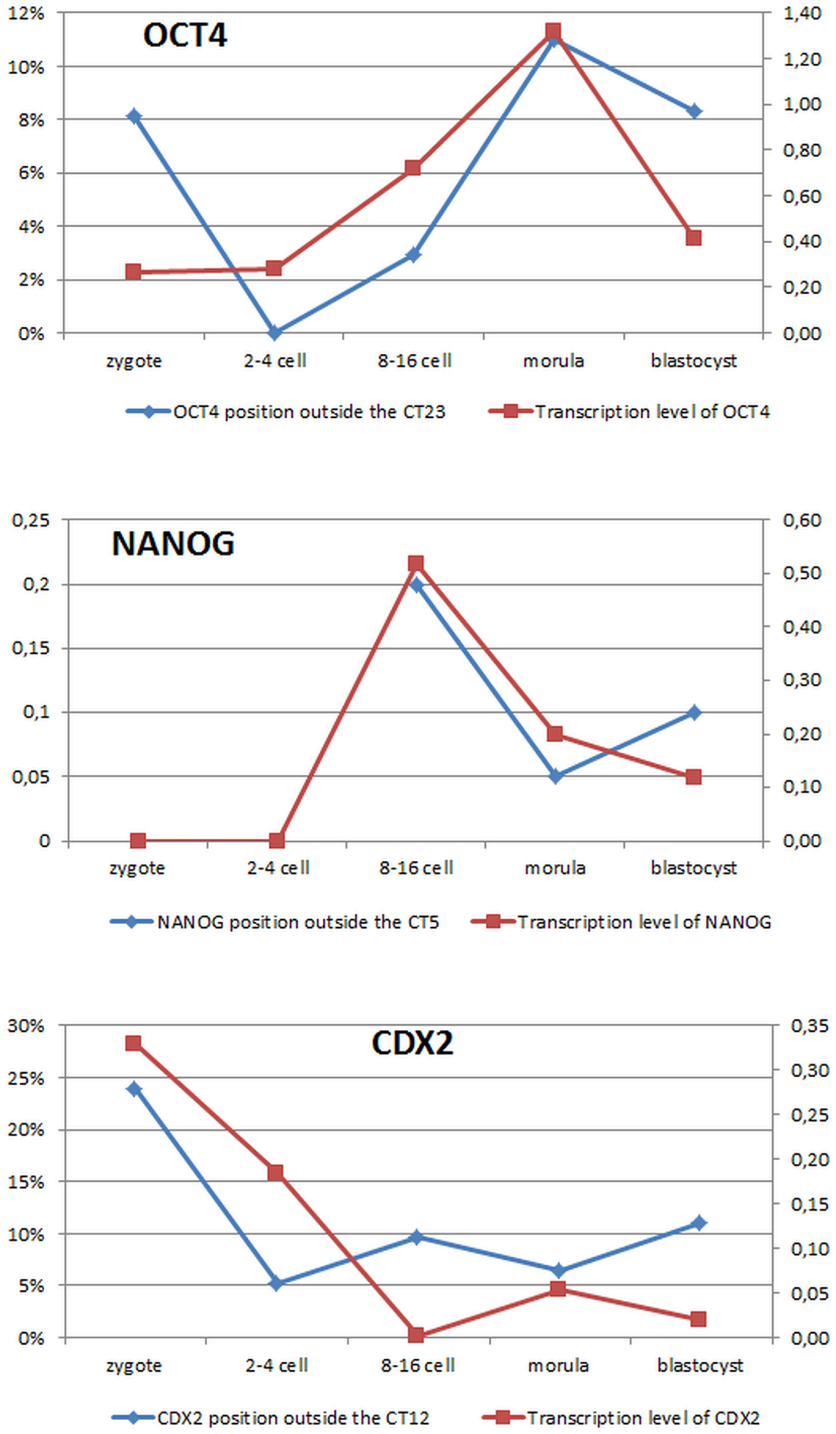
Changes in gene’s locus position correspond to its transcriptional activity. Y axis (left) presents the incidence (%) of gene locus moving away from its CT, and Y axis (right) indicates the relative transcript abundance. Because the quantitative gene expression studies were done on whole embryos (morula and blastocyst), the CT movement for the morula stage embryos has been presented as an average result obtained for morula inner and outer cells, and an average for ICM an TE for the blastocyst stage embryos.

The processes underlying early development are unique and as such may escape the generally accepted trend for somatic cells and embryo derived stem cells. Therefore, in conclusion we propose that (1) large chromosomes regardless of their gene density do not change radial position within the nucleus, irrespective of the developmental stage; (2) gene locus positon within the CT may be related to stage specific process of gene activation and lineage differentiation (high expression level—mRNA content, corresponds to locus moving away from its CT) (Fig 8) and (3) chromosome territories change their shape across developmental stages. As development progresses (differentiation is induced) changes in CT shape correlate to the developmental stage and stage specific gene expression pattern.

## Supporting information

S1 Fig Negative controls for immunofluorescence experiments. Example images of negative control experiments that were carried out for each immunolabelling procedure. Each time a cocktail of different secondary antibodies was used, the primary antibody was omitted. Arrows indicate oocyte nuclei. Symbols below the images represent the catalogue numbers of various secondary antibodies that were used (Santa Cruz Biotechnology, USA).
(TIF)

S2 Fig OCT4 distribution in bovine oocytes. Bovine immature oocytes (germinal vesicle stage, GV) and *in vitro* matured oocytes (MII stage) store OCT4 protein in the cytoplasm. α-Tubulin antibody (Sigma Aldrich, 096K4777) was used as a control of primary antibody penetration potential. Panel (A1) represents a cross section through the GV stage oocyte immunostained for OCT4 (green signal, Abcam, ab18976) and α-Tubulin (red signal). Arrow marks the nucleus. Panel (A2) shows different section of the same oocyte indicating the presence of α-Tubulin signal within the cytoplasm of immature oocyte. The images in (B) show MII stage oocyte with stage specific localization of α-Tubulin within the metaphase plate (marked by an asterisk) and a positive signal for OCT4 within the cytoplasm. Confocal sections were taken every 4μm. Chromatin was visualized by DAPI.
(TIF)

S1 Movie 3D projection of bovine morula stage embryo. An example of 3D projection of a morula stage embryo exhibiting positive fluorescent signals in all nuclei (DAPI staining, blue) for chromosome territories (CT12, red signal) and gene locus (CDX2, green signal).
(WMV)

S2 Movie 3D projection of irregular shaped chromosome territory (CT, red) and gene locus (green) localized outside CT. (WMV)

S3 Movie CT and gene locus distribution in bovine pronuclei. An example of 3D projection of zygote pronucleus (DAPI staining, blue) with peripherally distributed chromosome territory (CT, red) and gene locus (green) on the surface of CT. Z-stacks were captured with 0.5 μM interval.
(WMV)

S4 Movie CT and gene locus distribution in bovine trophectoderm. An example of 3D projection of trophectoderm cell nucleus (DAPI staining, blue) exhibiting intermediate localization of chromosome territory (CT, red) and gene locus (green) on the surface of CT. Z-stacks were captured with 0.4 μM interval.
(WMV)
