# Supplementary figures and images for: Changes in chromosome territory position within the nucleus reflect alternations in gene expression related to embryonic lineage specification
Source: PLoS One. 2017 Aug 2;12(8):e0182398. doi: 10.1371/journal.pone.0182398 (PMC5540545; doi:10.1371/journal.pone.0182398)

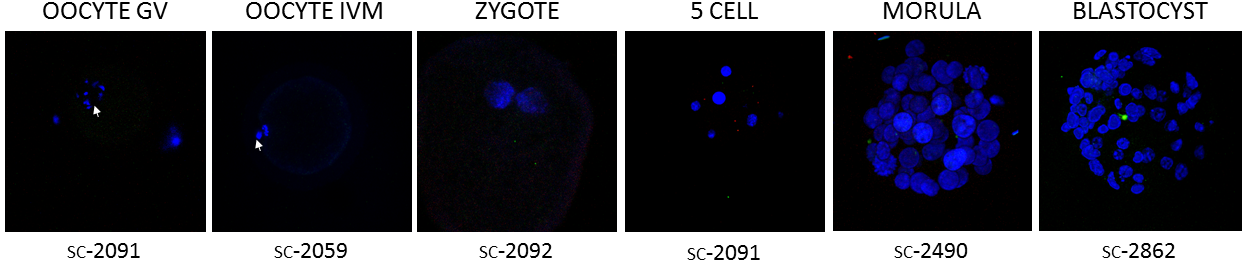

Supplement: S1 Fig — Example images of negative control experiments that were carried out for each immunolabelling procedure. Each time a cocktail of different secondary antibodies was used, the primary antibody was omitted. Arrows indicate oocyte nuclei. Symbols below the images represent the catalogue numbers of various secondary antibodies that were used (Santa Cruz Biotechnology, USA). (TIF) [file pone.0182398.s001.tif]

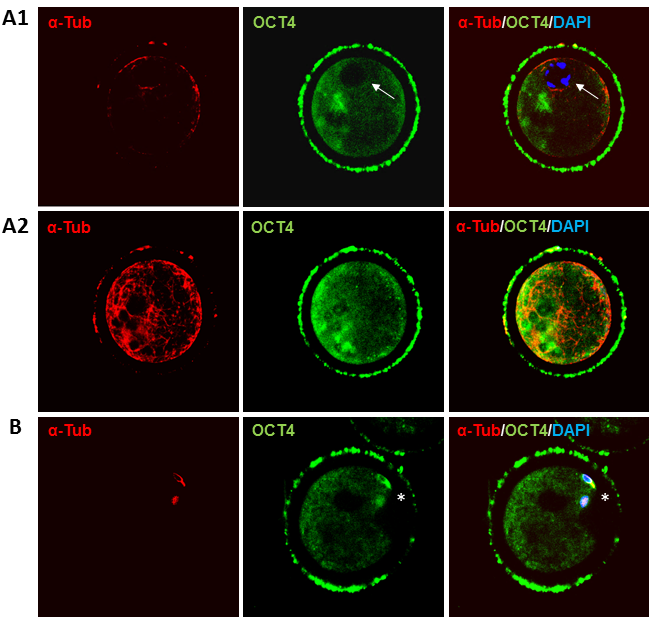

Supplement: S2 Fig — Bovine immature oocytes (germinal vesicle stage, GV) and in vitro matured oocytes (MII stage) store OCT4 protein in the cytoplasm. α-Tubulin antibody (Sigma Aldrich, 096K4777) was used as a control of primary antibody penetration potential. Panel (A1) represents a cross section through the GV stage oocyte immunostained for OCT4 (green signal, Abcam, ab18976) and α-Tubulin (red signal). Arrow marks the nucleus. Panel (A2) shows different section of the same oocyte indicating the presence of α-Tubulin signal within the cytoplasm of immature oocyte. The images in (B) show MII stage oocyte with stage specific localization of α-Tubulin within the metaphase plate (marked by an asterisk) and a positive signal for OCT4 within the cytoplasm. Confocal sections were taken every 4μm. Chromatin was visualized by DAPI. (TIF) [file pone.0182398.s002.tif]
